# Abrogation of Junctional Adhesion Molecule-A Expression Induces Cell Apoptosis and Reduces Breast Cancer Progression

**DOI:** 10.1371/journal.pone.0021242

**Published:** 2011-06-17

**Authors:** Masato Murakami, Costanza Giampietro, Monica Giannotta, Monica Corada, Ilaria Torselli, Fabrizio Orsenigo, Andrea Cocito, Giovanni d'Ario, Giovanni Mazzarol, Stefano Confalonieri, Pier Paolo Di Fiore, Elisabetta Dejana

**Affiliations:** 1 IFOM, Foundation FIRC Institute of Molecular Oncology, Milan, Italy; 2 Department of Biomolecular Sciences and Biotechnologies, School of Sciences, University of Milan, Milan, Italy; 3 Department of Medicine Surgery and Odontoiatrics School of Medicine, University of Milan, Milan, Italy; 4 European Institute of Oncology, Milan, Italy; University of Chicago, United States of America

## Abstract

Intercellular junctions promote homotypic cell to cell adhesion and transfer intracellular signals which control cell growth and apoptosis. Junctional adhesion molecule-A (JAM-A) is a transmembrane immunoglobulin located at tight junctions of normal epithelial cells of mammary ducts and glands. In the present paper we show that JAM-A acts as a survival factor for mammary carcinoma cells. JAM-A null mice expressing Polyoma Middle T under MMTV promoter develop significantly smaller mammary tumors than JAM-A positive mice. Angiogenesis and inflammatory or immune infiltrate were not statistically modified in absence of JAM-A but tumor cell apoptosis was significantly increased. Tumor cells isolated from JAM-A null mice or 4T1 cells incubated with JAM-A blocking antibodies showed reduced growth and increased apoptosis which paralleled altered junctional architecture and adhesive function. In a breast cancer clinical data set, tissue microarray data show that JAM-A expression correlates with poor prognosis. Gene expression analysis of mouse tumor samples showed a correlation between genes enriched in human G3 tumors and genes over expressed in JAM-A +/+ mammary tumors. Conversely, genes enriched in G1 human tumors correlate with genes overexpressed in JAM-A−/− tumors. We conclude that down regulation of JAM-A reduces tumor aggressive behavior by increasing cell susceptibility to apoptosis. JAM-A may be considered a negative prognostic factor and a potential therapeutic target.

## Introduction

JAM-A (Junctional adhesion molecule-A) is a small immunoglobulin expressed by different cell types including epithelial, endothelial cells, leukocytes, dendridic cells and platelets [1], [2], [3], [4]. Several studies, using blocking antibodies or genetically modified mice, documented a role of JAM-A in mediating neutrophil and monocyte infiltration in different experimental inflammatory conditions such as peritonitis, meningitis, liver and heart ischemia and others [1], [2], [3], [5], [6]. The mechanism of action of JAM-A in inflammation is complex and may be different depending on the cellular context.

In epithelial cells JAM-A is preferentially concentrated at tight junctions and cooperates with claudins in promoting cell to cell adhesion. In absence of JAM-A colonic mucosa epithelial cells looses permeability control, favoring inflammatory colitis [7], [8].

The role of JAM-A in tumor growth and dissemination is still a debated issue. In a recent work, we have crossed Rip1Tag2 mice (pancreatic islet tumor mouse model) with JAM-A null mice. Rip1Tag2 mice develop pancreatic tissue hyperplasia and highly vascularized adenoma which progress to invasive carcinoma [9]. In this particular model, tumor cells do not express JAM-A which is however present in the cells of the stroma. We observed a significant reduction of growth in JAM-A null mice due to increased immunological response of the host and decrease in angiogenesis. Conflicting data have been published on the role of JAM-A in breast cancer. Naik MU et al. [10] reported that JAM-A expression reduces breast cancer cell lines' invasion and motility *in vitro* and is inversely related to carcinoma aggressiveness and metastatic behavior in human patients. In contrast, McSherry et al. [11] using a larger clinical data set showed that JAM-A expression is a negative prognostic factor in breast cancer.

In the present paper we tackled the problem of the role of JAM-A in breast cancer by applying different experimental and complementary approaches. We examined mammary tumor growth and dissemination in JAM-A null mice crossed with mice expressing a mutant form of Polyoma virus middle T (PyVmT) under mammary tumor virus promoter (MMTV) [12]. We used tumor cells freshly isolated and cultured from MMTV-PyVmT mouse tumors or 4T1 mammary tumor cell line to understand the mechanism of action of JAM-A. Finally, we studied in a large group of human patients, whether JAM-A expression negatively or positively correlates with breast cancer progression.

Taken together data show that in absence of JAM-A tumors grow significantly less in MMTV-PyVmT mice. Consistently, we found an inverse correlation between JAM-A expression and cancer prognosis in human patients. *In vivo* studies of MMTV-PyVmT tumors and *in vitro* experiments on cultured tumor cells show that abrogation of JAM-A expression or function causes tumor cell apoptosis. This effect parallels altered organization of intercellular cell to cell junctions and may explain the decrease in tumor growth observed in absence of JAM-A.

## Materials and Methods

### Ethics Statement

Written informed consent for research use of biological samples was obtained from all patients, and the research project was approved by the Institutional Ethical Committee. Current Members of the IEO Ethics Committee: Luciano Martini (Chairman), Director of the Institute of Endocrinology, Milan; Apolone Giovanni (Vice Chairman), Chief of the Translational and Outcome Research Laboratory and the “Mario Negri” Institute, Milan; Bonardi Maria Santina, Head of the Nursing Service of European Institute of Oncology, Milan; Cascinelli Natale, Scientific Director of National Cancer Institute, Milan; Gallus Giuseppe, Director of Institute of Medical Statistics of Milan; Gastaldi Stefano, Psychologist and Psychotherapist, Scientific Director of Attivecomeprima; Goldhirsch Aron, Director of the Department of Medicine of European Institute of Oncology, Milan; La Pietra Leonardo, Chief Medical Officer of European Institute of Oncology, Milan; Loi Umberto, Export in Legal Procedures, Monza; Martini Luciano (Presidente), Director - Institute of Endocrinology, Milan; Merzagora Francesca, President of Italian Forum of Europa Donna, Milan; Omodeo Sale Emanuela, Director of Pharmaceutical Service, European Institute of Oncology, Milan; Pellegrini Maurizio, Head of the Local Health District, Milan; Rotmensz Nicole, Head of the Quality Control Unit, European Institute of Oncology, Milan; Tomamichel Michele, Director, Sottoceneri Sector Cantonal Sociopsychiatric Organisation, Lugano; Monsignor Vella Charles, Bioethicist and theologist, S. Raffaele Hospital and Scientific Institute, Milan; Veronesi Umberto, Scientific Director, European Institute of Oncology, Milan. OBSERVERS: Ciani Carlo, Chief Executive Officer, European Institute of Oncology, Milan; Della Porta Giuseppe, Research Co-ordinator, European Institute of Oncology, Milan; Michelini Stefano, Managing Director, European Institute of Oncology, Milan. SECRETERIAT OFFICE: Nonis Atanasio (head), Controlled Clinical Studies Office, European Institute of Oncology, Milan; Tamagni Daniela (Assistant), Controlled Clinical Studies Office, European Institute of Oncology, Milan.

### Tissue Samples

Formalin fixed and paraffin embedded specimens were provided by the Pathology Departments of the European Institute of Oncology (Milan).

#### Animal statement

Mice were housed according to the guidelines set out in Commission Recommendation 2007/526/EC - June 18, 2007 on guidelines for the accommodation and care of animals used for experimental and other scientific purposes. At the end of the experiment, mice were euthanized by inhalation of high concentrations of CO_2_. Currently, the Italian legislation does not require a specific ethical review process for all the experiments involving animals.

### Mammary Tumor and Lung Metastases

MMTV-PyVmT transgenic mice, a spontaneous mouse model of breast tumor were generated by WJ. Muller (McKMaster University, Ontario, Canada) [12] and provided by G. Christofori (University of Basel, Basel, Switzerland). They were crossed with JAM-A−/− mice to obtain a MMTV-PyVmT/JAM-A−/− strain. MMTV-PyVmT/JAM-A+/+ littermates were used as control. Mice were palpated twice a week and tumor appearance was recorded. Tumor-free survival was calculated from Kaplan–Meier curves and statistical significance was determined by the Log-Rank test for the survival studies and t-test for tumor growth studies. The metastatic ratio was evaluated by removing the lungs from anesthetized mice and inspecting metastatic nodules on the lung surface by using a stereoscope (Nikon SMZ800 stereoscope ×3–5, Tokyo, Japan) as described [13]. Tumor weight and lung metastasis logistic curve was made by JMP software (SAS Institute, Cary, North Carolina) and analyzed by Fisher's test.

### Immunofluorescence, Image Quantitative Analysis and Western blot

For the immune phenotypical characterization of MMTV-PyVmT/JAM-A+/+ and JAM-A−/− mice, tumor and lung samples or cultured cells were fixed in 4% paraformaldehyde or methanol for 24 h, and kept at 4°C in phosphate buffer saline (PBS) until use. 10 µm tissue sections and cell specimens were stained as previously reported [9], [14] using the following antibodies: CD4, CD8, CD11c, MHC-II, β-catenin (BD Bioscience, San Jose, CA), F4/80 (Serotec, Kidlington, UK), PECAM-1 (clone Mec13.3 or Millipore, Billerica, MA), Ki-67 (Abcam, Cambridge, MA), E-cadherin (Invitrogen, Carlsbad, CA), ZO-1, Cingulin (Zymed/Invitrogen, Carlsbad, CA), JAM-A (clone BV12), β1-integrin (Chemicon, Billerica, MA). TdT-mediated dUTP nick end labeling (TUNEL) staining was done using the In Situ Cell Death Detection Kit (Roche, Basel, Switzerland). Samples were analyzed under an AX-70 Provis (Olympus, Hamamatsu, Japan) fluorescence microscope equipped with a black and white cooled CCD camera (c5985, Olympus), or with a Leica TCS SP2 AOBS confocal microscope equipped with 405, 488, 543, and 633 nm laser lines (Wetzlar, Switzerland). Digital images were computer processed with Adobe Photoshop CS2 (Adobe Systems Inc, San Jose, CA). For the quantitative analysis of immunofluorescence experiments ImageJ image-analysis software (W. Rasband, National Institutes of Health) was used to measure the specific mean intensity on an average of ten cells for each antibody staining. Background intensity was initially subtracted by placing “regions of interest” over areas devoid of specific signal.

For Western blot analysis cells were lysed in Tris-buffer saline containing 0.1% Tween-20 (T-TBS) and 1% Triton X-100. Cell lysates were resolved by SDS-PAGE and transferred to nitrocellulose membranes (Millipore, Billerica, MA). Proteins of interest were visualized using specific antibodies, JAM-A (clone BV12/BV19/BV20), E-cadherin, (BD Bioscience), β1-integrin, ZO-1 (Cell signaling, Boston, MA), and ά-tubulin (Sigma, St Louis, MO), followed by peroxidase-conjugated secondary antibodies and by an enhanced chemiluminescence kit (Amersham Biosciences, Little Chalfort, UK).

### Cell Culture

4T1 mouse mammary tumor cells were obtained from the American Type Culture Company (Manassas, VA) and were cultured in RPMI 1640 medium (Gibco/Invitrogen, Carlsbad, CA) supplemented with 10% fetal bovine serum plus 1.0 mM sodium pyruvate, and 100 U/ml penicillin, and 100 µg/ml (Gibco/Invitrogen) as described [15]. JAM-A+/+, JAM-A−/− endothelial cells were isolated from the lung microcirculation and cultured as described [16]. To obtain primary tumor cells, late stage tumors (14–16 week old) from MMTV-PyVmT/JAM-A+/+ and JAM-A−/− females were harvested and minced using a razor blade, then digested using collagenase/hyaluronidase solution (Stem Cell Technologies, Vancouver, Canada) at 37°C for 2 hour. The samples were washed with 5 volumes of HBSS (Sigma)+2% fetal bovine serum and 2 mmol/L ethylenediaminetetraacetic acid (EDTA) (HFE) and centrifuged at 450×g for 5 min. The pellet was dissociated in 5 mL Trypsin-EDTA, then 2 mL dispase (Stem Cell Technologies): for 1 and 3 min respectively, by continuous pippetting, diluted with 10 mL HFE, and passed through a 40 µm cell strainer to obtain single-cell suspension. Isolation of epithelial cells was achieved through selective depletion of hematopoietic, endothelial, and stromal cells using a Mouse Epithelial Cell Enrichment Kit (Stem Cell Technologies). Briefly, the single-cell suspension was concentrated to1×10^8^ cells/mL. Biotinylated antibodies (0.5 µg per million cells) against stromal cells (CD140α, eBiosciences, San Diego, CA), endothelial cells (PECAM-1), and hematopoietic cells (CD45 and TER119) were added for 15 min at 4°C. Antibody conjugation to magnetic nanoparticles was achieved through 15-min incubation with EasySep Biotin Selection cocktail followed by 10 min with EasySep Magnetic Nanoparticles. An EasySep Magnetic device was used for 5 min to separate the non-epithelial cells that attached to the tube surface from the epithelial cells that remained in the supernatant. Mammary primary cells were cultured in 5% FBS and 10 ng/ml Epidermal Growth Factor (EGF) (Stem Cell Technologies), 10 ng/ml Fibroblast Growth Factor (FGF) and 4 µg/ml heparin containing complete EpiCult-B medium (Stem Cell Technologies). Non-attached cells and debris were flushed away the second day and the remaining cells were cultured in cytokine containing complete EpiCult-B medium.

### Cell Proliferation

4T1 cells were seeded and incubated for 24 hours in complete medium, followed by an overnight incubation in 10% FBS containing complete medium or in serum-free medium for 24, 48 or 72 hours. This was followed by fixation and staining with crystal violet. Stained cells were then solubilized with 10% acetic acid, and the 595 nm absorbance was measured for each time point.

### Wound Assay

4T1 cells were seeded, grown to confluence and starved overnight in the presence of 5 µg/ml control rat IgG antibody or BV11 JAM-A neutralizing antibody containing complete medium. Confluent monolayers were wounded with a pipette tip of approximately 1-mm of diameter. Cell monolayers were incubated with the antibody in complete medium and migration of 4T1 cells area was monitored by using phase contrast microscopy.

### Calcium Switch Assay

Confluent 4T1 cells were incubated with EGTA (2 mM in complete medium). 60 min later EGTA was aspirated and cells were incubated with control or BV11 antibody for 30, 60, and 120 min as described (19). At the end of each period of incubation cells were fixed and stained with anti E-cadherin antibody.

### Tissue-Micro Array (TMA)

All clinical investigation has been conducted according to Declaration of Helsinki principles. Large-scale TMA of 444 patient breast cancer case–control cohort studies were performed using human JAM-A antibody (R&D, Minneapolis, MN) as described [17].

### Statistical analysis

Disease-free period according to JAM-A expression was drawn using the Kaplan Meier method and compared by the Log-rank test. The correlation between the clinic pathological features of the tumors and JAM-A expression was evaluated; Odds ratio (OR) and 95% Confidence Intervals (CI) were obtained from logistic regression models using the JMP statistical software (SAS Institute, Inc., Cary, NC). A *p* value of less than 0.05 was considered to be statistically significant.

## Results

### MMTV-PyVmT/JAM-A−/− mice show decreased primary breast tumor growth

**Figure 1:**
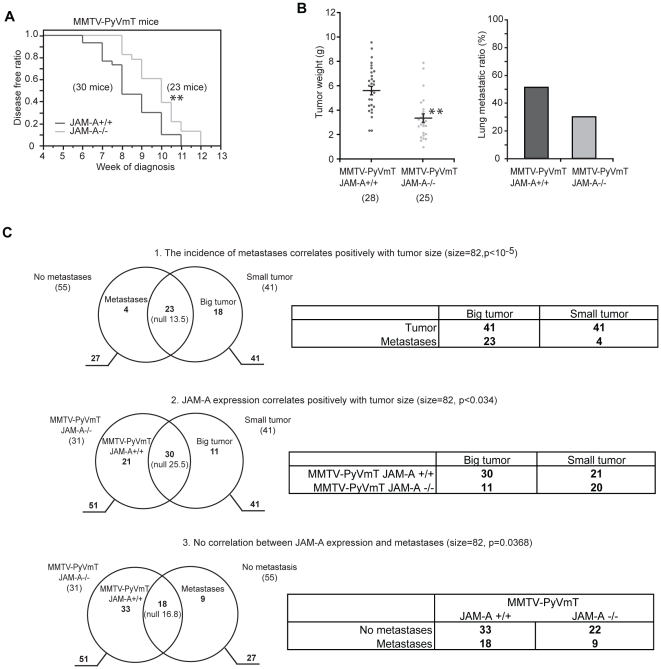
Genetic abrogation of JAM-A expression leads to reduction of mouse mammary tumor growth. A) MMTV-PyVmT/JAMA−/− mice had longer disease-free time compared to JAM-A+/+ mice; B) 13 week-old MMTV-PyVmT/JAM-A−/− mice present reduced primary tumor growth. Bars are means ± SEM. The lung metastases ratio (the percentage of mice with metastases out of the total number of mice with tumors) was higher in MMTV-PyVmT/JAM-A+/+ compared to JAM-A−/− mice but did not reach statistical significance (p = 0.091); C) Correlation between JAM-A expression, tumor size and lung metastases: The population was classified for tumor size in two groups on the basis of the median weight of tumors (3.55 g): big tumors (above median) and small tumors (below median), each composed of 41 mice; the population was then classified by expression of JAM-A (31 JAM-A −/− mice and 51 JAM-A +/+ mice) and by incidence of metastases (55 mice did not develop metastases and 27 did). C.1) Venn diagram of Big/Small (above or below median weight) tumors and metastases. There is a significant correlation between size of tumor and development of metastases (overlap between these two sets is 23 mice while the expectation from the null hypothesis would be 13.5, leading to a significant positive correlation by Exact Fisher Test with p<10^−5^). C.2) MMTV-PyVmT/JAM-A+/+ or JAM-A−/− and Big/Small tumors. Expression of JAM-A positively correlates with tumor size (out of the 51 JAM-A +/+ mice, 41 show big tumors and 30 are in the overlap of the two sets; significance of correlation p<0.034). C.3) MMTV-PyVmT/JAM-A+/+ or JAM-A−/− and metastases. The incidence of metastases is not significantly affected by JAM-A expression if balanced with tumor size. There is no significant correlation between expression of JAM-A (51 mice) and metastases (27 mice out of 82 mice), the overlap is 18 as shown in the Venn Diagram and the deviation from the expected 16.8 is not statistically significant (p = 0.368). All correlations are referred to a population of 82 animals, p Values are calculated by Exact Fisher Test, null values are the expectations for independent events.Tables on the right side of panel C summarize the results.

MMTV-PyVmT mice were interbred with JAM-A wild-type (MMTV-PyVmT/JAM-A+/+) or null mice (MMTV-PyVmT/JAM-A−/−) [12], [18]. JAM-A was expressed in the normal epithelium of mammary duct and glands in wild type mice and expression was maintained in MMTV-PyVmT mammary tumor cells and in metastatic tumor cells in the lymph nodes and lung (Figure S1). All MMTV-PyVmT/JAM-A+/+ and MMTV-PyVmT/JAM-A−/− mice developed mammary tumors within 12 weeks of age, consistent with previous reports [12]. However, tumors appeared earlier in MMTV-PyVmT/JAM-A+/+ mice compared to JAM-A−/− mice (8.53±0.26 weeks and 9.86±0.26 weeks respectively; p = 0.0048, Log-rank test) (Fig. 1A). At 13 weeks tumor size was significantly smaller in MMTV-PyVmT/JAM-A−/− than in MMTV-PyVmT/JAM-A+/+ mice (5.79±0.36 g versus 3.43±0.35 g p<0.01) (Fig. 1B left panel). Lung metastatic ratio (the percentage of mice with metastasis out of the total number of mice with tumors) was lower in JAM-A−/− than in JAM-A+/+ mice although the difference did not reach statistical significance (51.2% versus 30.0% Fisher's test p = 0.091) (Fig. 1B right panel).

More detailed mathematical analysis of the whole data set was performed (Fig. 1C). The population (82 mice) was classified for tumor size in two groups on the basis of the median weight of tumors (3.55 g): big tumors (above median) and small tumors (below median), each composed of 41 mice; it was then classified by expression of JAM-A (31 JAM-A −/− mice and 51 JAM-A +/+ mice) and by incidence of metastasis (55 mice did not develop metastasis and 27 did).

As expected there is a significant correlation between size of tumor and development of metastasis (overlap between these two sets is 23 mice while the expectation from the null hypothesis would be 13.5, thus leading of an indication of positive correlation that the Exact Fisher Test rates significant with p<10^−5^). Expression of JAM-A positively correlated with tumor size (out of the 51 JAM-A +/+ mice and 41 having big tumors 30 are in the overlap of the two sets, for a significance of the correlation with p<0.034), but there is no significant correlation between expression of JAM-A (51 mice) and metastasis (27 mice out of 82 mice), the overlap is 18 as shown in the Venn Diagram and the deviation from the expected 16.8 is not statistically significant (p = 0.368).

Logistic curve of mammary tumor size and metastasis also shows a positive correlation of tumor size and metastasis, but no significant correlation between incidence of metastasis and JAM-A expression (Figure S2). Taken together these results indicate that JAM-A expression positively correlates with MMTV-PyVmT mammary tumor growth while the incidence of metastasis was not specifically affected.

### JAM-A expression increases tumor cell apoptosis

**Figure 2:**
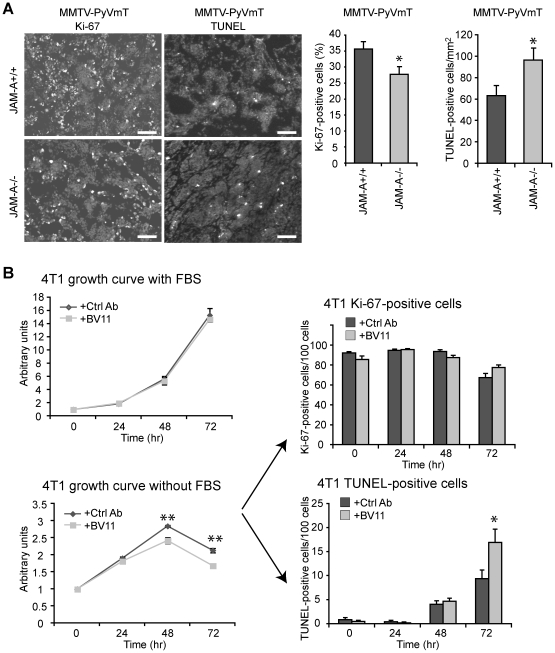
Abrogation of JAM-A expression increases tumor cell apoptosis. A) Histological examination shows lower cell proliferation and higher apoptosis in MMTV-PyVmT/JAM-A−/− as compared to JAM-A+/+ tumors. Right panels show quantification of Ki-67 or TUNEL positive cells. Data are means +/− SEM *p<0.05 by unpaired Student's t test. Scale bar: 100 µm. B) The growth curve of 4T1 cells in the presence of a control (Ctrl) antibody (Ab) or a JAM-A neutralizing Ab (BV11) was measured in the presence (left upper panel) or absence of fetal bovine serum (FBS) (left lower panel). Under starving conditions, upon exposure to a JAM-A blocking Ab the total cell number was significantly reduced while the number of TUNEL-positive cells was increased. Values are means ± SEM of at least 4 replicates from 3 independent experiments. *p<0.05, **p<0.01 by unpaired Student-t test.

JAM-A is expressed in mammary tumor vasculature (Figure S1). We have previously shown that the absence of JAM-A reduced Rip1Tag2 pancreatic islet tumor size, angiogenesis and inflammatory infiltrate [9]. Analysis of MMTV-PyVmT tumors showed that in absence of JAM-A, tumor cells proliferate slightly less (27.73±2.29% versus 35.6±2.02, p<0.05) but undergo apoptosis to higher extent than in the presence of this adhesive molecule (96.4±10.2 versus 63.1±8.5 cells per mm^2^ p<0.05) (Fig. 2A). Conversely, no statistically significant difference in tumor angiogenesis (Figure S3A), inflammatory (F4/80 positive cells, Figure S3B) and immune cell infiltrate (CD11c and MHCII positive cells) was observed (Figure S3C). The number of CD4+ and CD8+ lymphocytes was increased in the tumors but the difference did not reach statistical significance.

*In vitro* incubation of BV11 JAM-A neutralizing Ab [19] with JAM-A positive 4T1 cell line [15] (Figure S4A) did not change cell proliferation in complete medium but significantly reduced the cell number in absence of serum (Fig. 2B and C). In starvation, TUNEL-positive cells were markedly increased (9.34±1.80 versus 16.9±2.75%, p = 0.03) (Fig. 2E) suggesting that the decreased number of cells is due to apoptosis. However, the number of 4T1 cells treated with BV11mAb was lower already at 48 hours when TUNEL staining was not yet apparent. It is likely, as shown by others [10], [20], that JAM-A −/− cells may detach from the plate before showing signs of apoptosis or, alternatively, that besides its antiapoptotic activity, JAM-A is required for correct tumor cell growth.

### JAM-A affects MMTV-PyVmT mammary cell morphology and cell-to-cell junction

**Figure 3:**
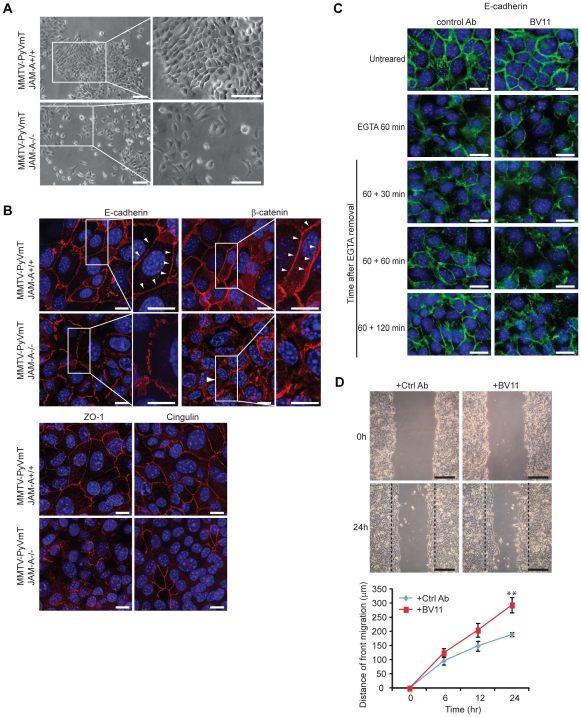
Inhibition of JAM-A expression and function affects mammary tumor cell morphology and organization of cell-cell junctions. A) Phase-contrast microscopy of cultured mammary tumor cells derived from MMTV-PyVmT/JAM-A+/+ and JAM-A−/− tumors. JAM-A+/+ cells formed confluent islands with epithelioid morphology and tight intercellular junctions. In contrast, JAM-A−/− cells form loosely organized islands and frequently grow as single cells with spindle shape morphology. Scale bar; 200 µm; B) Immunofluorescence staining of markers of adherens junctions (E-cadherin, β-catenin) and tight junctions (ZO-1, cingulin). While JAM-A+/+ tumor cells present a continuous junctional staining of E-cadherin, β-catenin, ZO-1 and cingulin at intercellular contacts, in JAM-A−/− cells the distribution of these markers was reduced or discontinuous (arrowhead). Scale bar; 15 µm; C) The effect of BV11 JAM-A blocking Ab on Ca^2+^ switch assay. 4T1 cells were incubated with EGTA for 60 min to disrupt E-cadherin junctional localization and Ca^2+^ was then added back to the cells to restore E-cadherin staining. In the presence of a JAM-A blocking Ab BV11 E-cadherin staining was partially inhibited and still largely incomplete up to 120 min. Scale bar; 20 µm; D) 4T1cell migration was significantly increased by a JAM-A blocking Ab (BV11). Upper panels report phase contrast microscopy of a typical experiment. Scale bar; 500 µm. Lower panels show a quantification of the distance of migration of the front. Values are means ± SEM of 4 replicates from a typical experiment out of 3 performed. **p<0.01 by unpaired Student-t test.

We then cultured tumor cells from MMTV-PyVmT/JAM-A+/+ and JAM-A−/− tumors. JAM-A positive cells show a correct epithelioid morphology while JAM-A null cells showed spindle or round shape morphology and weak cell to cell adhesion (Fig. 3A). At immunofluorescence analysis MMTV-PyVmT/JAM-A−/− cells presented partially disorganized adherens junctions with discontinuous staining of E-cadherin and β-catenin, and loss of tight junction markers such as ZO-1 and Cingulin in many areas of the monolayer (Fig. 3B). However, no significant change in the total amount of junctional markers and other adhesive and cytoskeletal proteins (E-cadherin, β-catenin, cingulin, ZO-1, vinculin and β1 integrins) was observed in the presence or absence of JAM-A (Figure S4B and C).

Interestingly, sections of mammary glands of JAM-A +/+ and −/− mice presented partly disorganized E-cadherin staining suggesting that, also in the absence of tumor, JAM-A expression is required for a correct epithelial junction organization (Figure S5). TUNEL staining was not significantly increased in normal JAM-A −/− mammary glands. Possibly, additional factors such as hypoxia may contribute to tumor cell apoptosis in absence of JAM-A.

In a Ca^2+^ switch assay the presence of BV11 mAb significantly prolonged the time of junction recovery (complete recovery of E-cadherin staining could be observed within 60 min in control antibody treated cells while BV11 treated cells showed only partial recovery up to 120 min) as reported [21] (Fig. 3C).

A further evidence of the role of JAM-A in the correct organization of cell-to-cell junctions is given by the wound assay (Fig. 3D). In this assay the weaker is cell-to-cell adhesion at junctions, the higher is capacity of cells to migrate into the wound [22]. As reported in the figure, when JAM-A was blocked by BV11 mAb cells migrate into the wound significantly more efficiently.

**Figure 4:**
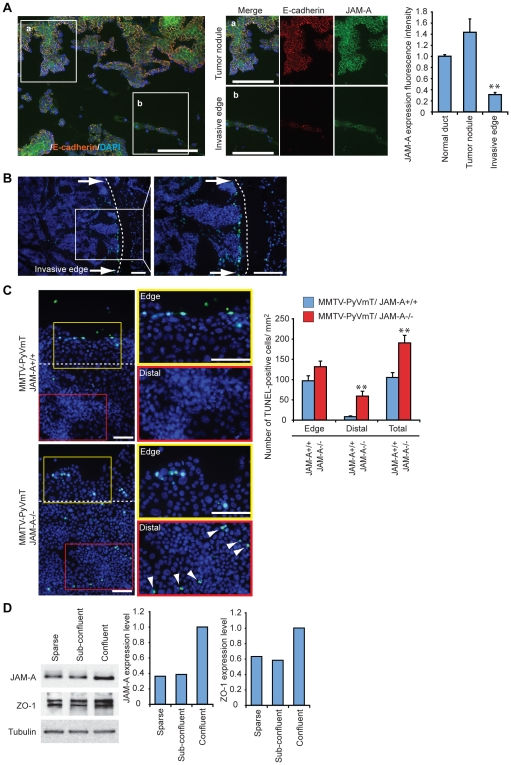
JAM-A and junctional proteins' expression was decreased at the invading tumor edge in vivo and in vitro. A) MMTV-PyVmT/JAM-A+/+ tumor sections show decreased JAM-A and E-cadherin expression level at the invasive edge as compared to tumor nodules and mammary duct. Scale bar; 100 µm; B) In tissue sections, TUNEL positive tumor cells were concentrated at the invasive edge of the tumor. Scale bar; 100 µm; C) In the wound assay (see Fig. 3) cultured MMTV-PyVmT/JAM-A−/− cells show strong TUNEL staining both at the migrating front and in the distal areas while JAM-A+/+ cells presented TUNEL staining only at the leading edge. Quantification is reported at the right panel, data are means +/− SEM of 3 experiments performed in quadruplicates. **p<0.01 by unpaired Student-t test. Scale bar; 300 µm; D) Western blot analysis of JAM-A and ZO-1 expression in 4T1 cells in sparse, sub-confluent, and confluent conditions. Quantification is reported in the right panels. The results are representative of 3 independent experiments.

These data were further supported by *in vivo* observations. In MMTV-PyVmT carcinomas (Fig. 4A) we found that JAM-A is strongly decreased at the tumor invasive edge as compared to tumor nodules (Fig. 4A, compare a and b). The decrease in JAM-A and ZO-1 could be seen also in sparse/subconfluent cell cultures as compared to confluent cells (Fig. 4D). Consistently, when cells invade the surrounding tissue and junctions are partially dismantled, JAM-A expression is significantly reduced.

We then asked whether there is a correlation between the effect of JAM-A on junctions and its anti apoptotic activity. Invading tumor cells in a wounded monolayer *in vitro* or at the invading edge of a tumor *in vivo* were positive to TUNEL staining (Fig. 4B and C) This marginal zone corresponds to the area where JAM-A is reduced. Most importantly, the number of TUNEL positive cells was strongly increased in JAM-A−/− tumor cells, not only at the leading edge but also in the more internal regions of the monolayer (Fig. 4D). In contrast JAM+/+ cells were apoptotic only at the migrating rim while in the internal regions of the monolayers, where JAM-A is correctly expressed and organized at junctions, cells were TUNEL negative.

Thus, as previously described in other cell types [10], [11], [23], JAM-A is required for correct junction organization in mammary tumor cells. Stable junctions are known to protect the cells from apoptotic stimuli [24], when JAM-A is reduced or junctions are dismantled, as in migrating cells or in JAM-A null tumor cells, apoptosis is increased.

### Trascriptomic analysis of MMTV-PyVmT/JAM-A−/− or JAM-A+/+ mammary tumor and gene-set enrichment analysis (GSEA)

To investigate the phenotype of JAM-A positive and null tumors we compared gene profiles of early stages of MMTV-PyVmT/JAM-A+/+ and JAM-A−/− tumor extracts. We obtained a gene list of total 547 differentially expressed genes (pset) (the “MMTV-PyVmT/JAM-A−/− signature”) presented in Table S1 Sheet 1. “MMTV-PyVmT/JAMA−/− signature” genes were classified by functional and network annotation by Ingenuity Pathways Analysis (IPA) program and top-ranked classification of the differentially expressed genes was performed. The functional classification indicates that genes involved in apoptosis, cell morphology, cell growth and movement are grouped at the top of the list (Table S1, Sheet 2 “Functional classification” and Sheet 3 “Network Classification”). We then analyzed GSEA by using MMTV-PyVmT/JAM-A−/− signature and G1–G3 human breast tumor published data set derived from the studies of Ivshina et al [25]. Interestingly, from GSEA analysis we found that the genes over expressed by MMTV-PyVmT/JAM-A+/+ mice tumors are also over expressed in human G3 tumors, whereas genes over expressed by JAM-A−/− mice tumors are over expressed in human G1 tumors (Table S2, Sheet 1 “GSEA Dataset G3 vs G1” and Sheet 2 “GSEA Dataset G3 vs G1 overlap”).

### JAM-A is a negative prognostic factor in human breast tumor patients

**Figure 5:**
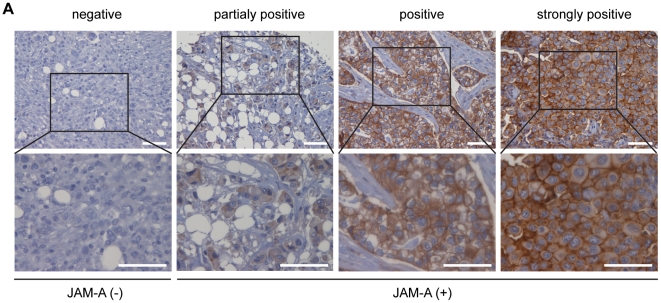
Association of JAM-A expression and prognosis in clinical samples. A) A total 444 human breast cancer were examined. Scores were evaluated on the level of JAM-A expression at cell membrane. Tumors were divided in *JAM-A negative* when no JAM-A was detected and *JAM-A positive* when JAM-A staining, either complete or incomplete, was present on the membrane of more than 10% of tumor cells. Scale bar; 200 µm.

Expression JAM-A was determined in a case-control study composed of a total of 444 breast tumor samples, arrayed into 5 different TMAs (Table S3) [17]. We classified the tumors in two major groups for expression of JAM-A: negative (tumors where JAM-A is absent) versus positive (tumors with different degrees of JAM-A expression (Fig. 5A). Clinico-pathological associations are summarized in Table 1. JAM-A expression positively correlates with tumor stage (p = 0.002), nodal status (p = 0.001), Elston Grade (p = 0.024) and Poor Prognosis Group according to the Nottingham Prognostic Index (NPI) (p<0.001); Ductal Tumors tend to form glandular structures, whereas lobular tumors are less cohesive and invade in single file. In line with this notion, JAM-A expression is frequently abrogated in Lobular Breast Carcinomas in comparison to Ductal Carcinomas (p = 0.007). In univariate analysis, JAM-A expression significantly correlated with a higher risk of developing a breast-related event (Table 2). However in a multivariate analysis in which all events were evaluated in relation to relevant prognostic factors, such as pathological stage, tumor grade, estrogen receptor expression, nodal status, Ki67 and Her2/Neu expression, JAM-A lost its predictive power (Table 2).

**Table 1:** Correlation of JAM-A expression and clinicopathological parameters in invasive breast carcinomas.

JAM_A	NEG	POS	OR (C.I.)	Pvalue
**PATIENTS**	**273**	**135 (33.1%)**		
**AGE**				
<50	126	70 (35.7%)		
> = 50	147	64 (30.3%)	0.784 ( 0.517–1.185 )	0.249
**HISTOTYPE**				
Ductal	216	**120 (35.7%)**		
Lobular	51	11 (17.7%)	0.388 ( 0.186–0.747 )	**0.007**
**pT**				
1	162	59 (26.7%)		
2–4	99	**70 (41.4%)**	1.941 ( 1.268–2.984 )	**0.002**
**LN**				
Neg	160	54 (25.2%)		
Pos	113	**80 (41.5%)**	2.098 ( 1.381–3.207 )	**0.001**
**GRADE**				
G1	57	16 (21.9%)		
G2	102	50 (32.8%)	1.417 ( 1.041–1.945 )	0.093
G3	78	47 (37.6%)	2.009 ( 1.084–3.786 )	**0.024**
**ER**				
<10%	72	45 (38.5%)		
> = 10%	188	84 (30.9%)	0.715 ( 0.455–1.128 )	0.146
**PGR**				
<10%	99	62 (38.5%)		
> = 10%	155	67 (30.2%)	0.69 ( 0.45–1.059 )	0.089
**ERBB2**				
Neg	215	106 (33%)		
Pos	32	24 (42.9%)	1.521 ( 0.847–2.705 )	0.155
**Ki67**				
<16%	122	55 (31.1%)		
> = 16%	132	74 (35.9%)	1.244 ( 0.812–1.911 )	0.317
**NPI**				
GPG	87	23 (20.9%)		
MPG	91	46 (33.6%)	1.912 ( 1.079–3.459 )	0.029
PPG	44	**38 (46.3%)**	3.267 ( 1.75–6.219 )	**<0.001**

TMA (Immunohistochemical-Tissue Micro Array (IHC-TMA). Odds ratio (OR) and 95% Confidence Intervals (CI) were obtained from logistic regression models. Note that the number of scored cases is lower that the total number of cases since: i) in some cases, individual cores detached from the slides during the manipulations; ii) clinical information was not available for all patients. In tumor tissues the IHC signals were associated with the tumor cell component and not with the adjacent or infiltrating stroma. Nottingham Prognostic Index (NPI) combines nodal status, tumor size and histological grade. According to NPI's score patients can be divided into 3 class: Good Prognosis Group (GPG), Moderate Prognosis Group (MPG) and Poor Prognosis Group (PPG). Primary tumor size according to the TNM staging system, pT; Estrogen receptor, ER; Progesterone receptor, PGR. LN: Lymph Node status.

**Table 2:** Univariate and multivariate analyses of JAM-A expression in breast cancer case-control datasets.

CASE-CONTROL COHORT (N = 444)				
ALL EVENTS	Univariate		Multivariate	
	*OR (95% CI)	*P*	*OR (95% CI)	*P*
**JAM-A Pos. vs. Neg.**	**1.593** (1.05–2.42)	**0.028**	1.474 ( 0.81–2.69 )	0.205

DISTANT EVENTS	Univariate		Multivariate	
	*OR (95% CI)	*P*	*OR (95% CI)	*P*
**JAM-A Pos. vs. Neg.**	1.416 (0.86–2.31)	0.165	1.557 ( 0.76–3.19 )	0.224

All events, loco-regional relapse, distant metastasis or controlateral breast cancer; distant events, distant metastasis. Odds ratio (OR) and 95% Confidence Intervals (CI) were obtained from logistic regression models and adjusted for age, pathological stage, histotype, tumor grade, hormone-receptor status, nodal status, Ki-67, NPI and ErbB2 expression in the multivariate analysis.

## Discussion

The results presented here show that JAM-A is a negative prognostic factor for murine and human mammary tumor growth. This conclusion is supported by a different and complementary set of results. In MMTV-PyVmT mammary tumor mouse model, genetic ablation of JAM-A leads to a significant delay in tumor appearance and growth. The number of metastasis tends to be lower in absence of JAM-A, although the difference is not significant (Fig. 1). Since the incidence of metastasis correlates with tumor size, it is likely that the lower incidence of metastasis is a consequence of a smaller tumor size.

In a previous work we found that the absence of JAM-A inhibited the growth of Rip1Tag2 carcinoma [9]. This effect was mediated by the immune reaction of the stroma since these particular tumor cells do not express JAM-A. However, in MMTV-PyVmT tumor model the immune and inflammatory cell infiltrate was very poor and, in absence of JAM-A, we could not observe significant differences in dendritic cell and monocyte infiltration. The number of infiltrating CD4+ and CD8+ cells was increased in JAM-A null tumors but the difference was not statistically significant. Overall, we cannot exclude that the immune reaction may contribute to reduced tumor progression in JAM-A−/− mice but it is hard to attribute only to this effect the observed reduction in tumor size. In contrast to Rip1Tag2, MMTV-PyVmT tumor cells are positive for JAM-A. Normal mammary gland and ductal epithelium express relatively high amounts of JAM-A (Figure S1) which is maintained high in tumor cells. We found that in MMTV-PyVmT/JAM-A−/− tumors and tumor cells derived from them, apoptosis was markedly increased. The anti apoptotic effect of JAM-A was also apparent in the mouse breast tumor cell line 4T1 when JAM-A was blocked by a specific antibody.

An important issue is the definition of the mechanism of action of JAM-A in protecting tumor cells from apoptosis. A correct organization of cell to cell junctions induces cell resistance to apoptotic stimuli through activation of different pathways [24], [26], [27], therefore agents able to dismantle intercellular junctions increase cell sensitivity to apoptosis. We report that JAM-A disrupts junction architecture. Furthermore, apoptosis was increased at the JAM-A+/+ tumor invasive front *in vivo* where JAM-A expression is also decreased. Consistently, genetic ablation of JAM-A increased apoptosis not only at the invasive front but also diffusely in all tumor cells.

The block of JAM-A decreases 4T1 cell number at 48 hours when the difference in apoptosis is still undetectable. The reduction in cell number is apparent only in absence of serum but not when cells are grown in complete medium (Fig. 2B and C). This suggests that inhibition of JAM-A may reduce cell growth when cells are in starvation.

However, since at 48 hours of starvation cells also show signs of partial retraction we cannot exclude that the observed reduction in cell number is due to cell detachment. Other authors have shown that, when JAM-A expression or activity is reduced in cultured mammary tumor [10] and renal carcinoma cells [20], the cells detach more easily from a monolayer and migrate more efficiently. These observations have been interpreted as increased tumor cell invasive and metastatic capacity but direct *in vivo* data in preclinical models have not been reported in these papers. As shown here the lack of JAM-A and the consequent alterations in junction organization leads to increased cell migration but also sensitivity to apoptosis. The final balance of these two aspects of JAM-A activity may determine the overall tumor cell dissemination *in vivo*.

In the epithelium of the mouse mucosa abrogation of JAM-A expression increased permeability and cell sensitivity to pro apoptotic inflammatory stimuli. In these cells the absence of JAM-A did not severely affect junctions organization in normal conditions but exacerbated the effects of inflammatory agents in bowel disease [7], [8]. As reported here, in normal mammary glands and more dramatically in breast cancer cells, in absence of JAM-A, junctions were altered. Cancer cell junctions are relatively poorly organized and it is not surprising that the decrease/abrogation of expression of a junctional component, such as JAM-A, induces more dramatic effects than in normal epithelial cells.

The data presented here using a preclinical model are consistent with the results obtained by TMA analysis of a group of 444 patients' specimens. Through this analysis we found a significant correlation between JAM-A expression and a poor tumor prognosis. McSherry et al. [11] observed a similar type of correlation in a group of 270 patients with invasive breast cancer, while Naik et al. [10] found an opposite correlation between JAM-A expression and tumor invasion in 12 tumors and their corresponding non neoplastic tissue as well as 50 malignant and their corresponding metastatic lymph node samples. Taking into consideration the larger data set of McSherry et al. [11], the present work (270 and 444 patients respectively) and the association of clinicopathological data, we feel rather confident that in a majority of cases high JAM-A expression is a prognostic factor of a poor patient outcome.

A further support to this conclusion comes from GSEA. Comparison of gene expression profiles of JAM-A+/+ and −/− tumors and the G1–G3 human breast tumor published data [25] shows that genes over expressed by MMTV-PyVmT/JAM-A+/+ mice tumors are also over expressed in human G3 tumors, whereas genes over expressed by JAM-A−/− mice tumors are over expressed in human G1 tumors. This analysis further supports the concept that JAM-A expression is associated with a “poor prognosis signature”. Interestingly, essentially no overlapping between G1 and G3 genes was observed comparing JAM-A+/+ and −/− tumors.

McSherry et al. [11], [28] and Gotte et al. [29] found that mammary tumor cell lines have a reduced motility when JAM-A is low and this effect was attributed to a lower expression of β1 integrins [11], [28]. Integrins can also transfer anti apoptotic signals and we cannot exclude that changes in β1 integrin expression could further contribute to JAM-A mediated anti apoptotic activity. In the present work in both MMTV-PyVmT derived tumor cells or 4T1 cell lines we observed increased and not decreased migration and we did not detect a significant change in β1 expression levels (Figure S4B). This discrepancy may be easily attributed to the different cell lines used and, possibly, to a different set of integrins expressed [18], [19], [22].

In conclusion, *in vitro* and *in vivo* data, using a genetic mammary tumor model or analyzing a large group of patient specimens, are all consistent with a negative prognostic role of JAM-A expression in breast cancer. We propose that this effect is due, at least in part, to the protective effect of JAM-A on tumor cell apoptosis and is likely correlated to disruption of a correct junction organization. The mechanisms through which tumor cells maintain high JAM-A during tumor progression remains to be defined. However, a recent publication [29] shows that JAM-A is a target for miR-145 which, in turn, is downregulated in breast cancer cells. These data may, therefore, explain the high levels of JAM-A in this particular type of tumor.

## Supporting Information

Figure S1 **JAM-A is expressed both in wild type and MMTV-PyVmT mice mammary tumors by immunohistological analysis of tissue samples.** (A) JAM-A is expressed in epithelial of JAM-A +/+ mammary gland mice (left panel) and not in that of JAM-A −/− mice (right panel). Scale bar 200 µm. (B) JAM-A is expressed in epithelial of normal mammary gland (left panel) and MMTV mammary tumor (right panel). Scale bar 100 µm. (C) JAM-A is expressed in primary mammary tumor, metastatic lymph nodes and metastatic lung tumor. Scale bar 400 µm.
(TIF)

Figure S2 **Mammary tumor weight positively correlates with lung metastasis in MMTV-PyVmT mice, but JAM-A expression does not affect this parameter.** Relationship between primary mammary tumor size and lung metastases of MMTV mice with or without JAM-A. Primary mammary tumor weight and lung metastases are positively correlated both in MMTV-PyVmT/JAM-A+/+ and in MMTV-PyVmT/JAM-A−/− mice but the lung metastatic ratio show no significant difference between these groups.
(TIF)

Figure S3 **Immunohistological analysis of MMTV-PyVmT/JAM-A−/− or JAM-A+/+ tumors.** A) Vascular density evaluated by PECAM-1 staining of vessels was not significantly different in JAM-A +/+ and −/− tumors (46252±2776 versus 49838±4311 µm^2^/mm^2^ p = 0.488; means ± SEM) Scale bar; 100 µm. B) No significant difference were observed in infiltration of CD11c- and MHC-II-positive dendritic cells, F4/80-positive macrophages and CD4- and CD8-positive leukocytes in MMTV-PyVmT/JAM+/+ or −/− tumors. (CD11c-positive cells, 723±49.33 vs 674.47±49.08 cells per mm^2^ p = 0.489; MHC-II-positive cells 417.8±31.12 vs 423.73±48.03 cells per mm^2^ p = 0.918; F4/80-positive cells 643.73±43.61 vs 669.27±42.40 cells per mm^2^ p = 0.677; CD4-positive cells 46.2±5.84 vs 64.8±8.58 cells per mm^2^ p = 0.080; CD8-positive cells 20.33±3.60 vs 28.87±4.69 cells per mm^2^ p = 0.156. Data represent means ± SEM).
(TIF)

Figure S4 **Characterization of mammary cells.** A) JAM-A expression in 4T1 cells by Western blot analysis. Data show that 4T1 cells express a comparable level of JAM-A as compared to wild type endothelial cells (EC). The specificity of the Ab (BV11) was indicated by lack of staining of EC from JAM-A−/− mice [18]. B) Western blot analysis shows that cultured MMTV-PyVmT/JAM-A+/+ mammary tumor cells express JAM-A in sparse and confluent conditions. As expected, JAM-A staining was absent in cells derived from tumors of JAM-A null mice. Integrin β1 chain or E-cadherin were not significantly changed in the presence or absence of JAM-A.
(TIF)

Figure S5 **Inhibition of JAM-A expression affects the organization of cell-cell junctions of mammary gland, but does not affect apoptosis.** A) Immunofluorescence staining of E-cadherin and TUNEL. While in JAM-A+/+ mammary glands epithelial cells present a continuous junctional staining of E-cadherin at intercellular contacts (a, a″), in JAM-A−/− mammary glands the distribution of this marker was more discontinuous (b, b″). No differences of the TUNEL staining have been observed (a′, b′). Scale bars: 100 µm (a, a′,b, b′) and 10 µm (a″, b″).
(TIF)

Table S1 **Characterization of MMTV-PyVmT JAM-A−/− signature.** “MMTV-PyVmT JAM-A-signature” sheet: This file lists the probesets of the MMTV-PyVmT/JAM-A−/− signature. For each entry, the following information is reported (from left to right): (1) probeset (corresponding to the detecting probeset on Affymetrix GENE 1.0 ST Mouse GeneChips), (2) GenBank accession number of the corresponding gene, (3) Gene Symbol, (4) regulation, the criteria to designate a probeset as JamA− (MMTV-PyVmT/JAM-A−/−) up or down versus JamA+ (MMTV-PyVmT/JAM-A+/+). Genes were considered upregulated (upregulated in MMTV-PyVmT/JAM-A−/−, highlighted in red) or downregulated (upregulated in MMTV-PyVmT/JAM-A+/+, highlighted in green), (5) FC (fold-change) in the JamA-/JamA+ comparison, across all samples, (6) P-value of FC in the JamA-/JamA+, (7) Description of gene. “Functional classification” sheet: This file reports the functional annotation of genes of the MMTV-PyVmT JAM-A-signature. “Network classification” sheet: This file reports the network annotation of genes of the MMTV-PyVmT JAM-A-signature.
(XLS)

Table S2 **“GSEA Dataset G3 vs G1” sheet: Genes of the overlap between MMTV-PyVmT JAM-A-signature and enriched genes in G3 versus G1 tumors of Ivshina data set.** For each entry, the following information is reported (from left to right): (1) Mouse Gene Symbol from MMTV-PyVmT JAM-A- signature, (2) GenBank accession number of the corresponding gene, (3) Ratio in the JamA-/JamA+, (4) FC (fold-change) in the JamA−/JamA+ comparison, (5) P-value of FC in the JamA−/JamA+ (6) Human Symbol (Ivshina data set corresponding to the detecting probeset on Affymetrix GENE 1.0 ST Mouse GeneChips) (7) U133 Probe Set (of Ivshina affimetrix HG-U133 Human GeneChips), (8) GSEA ENRICHMENT, genes with a “Yes HIGH in G1” gene in high expression in G1 breast tumor or a “Yes HIGH in G3” gene in high expression in G3 breast tumor. “GSEA Dataset G3 vs G1 overlap” sheet: Selected from overlap genes between MMTV-PyVmT JAM-A- signature and G3 or G1 upregulated genes from sheet “GSEA Dataset G3 vs G1”.
(XLS)

Table S3 **Clinical and pathological information of the case-control dataset of breast cancer patients.** The clinical and pathological information of the patients of the case-control dataset is reported. Disease recurrence (all events and distant events) was within 7 years. For some patients not all information was available. Nottingham Prognostic Index (NPI) combines nodal status, tumor size and histological grade. According to NPI's score patients can be divided into 3 classes: Good Prognosis Group (GPG), Moderate Prognosis Group (MPG) and Poor Prognosis Group (PPG). Primary tumor size of WHO classification, pT; Estrogen receptor, ER; Progesterone receptor, PGR.
(DOC)
