# Supplementary figures and images for: Abrogation of Junctional Adhesion Molecule-A Expression Induces Cell Apoptosis and Reduces Breast Cancer Progression
Source: PLoS One. 2011 Jun 17;6(6):e21242. doi: 10.1371/journal.pone.0021242 (PMC3117883; doi:10.1371/journal.pone.0021242)

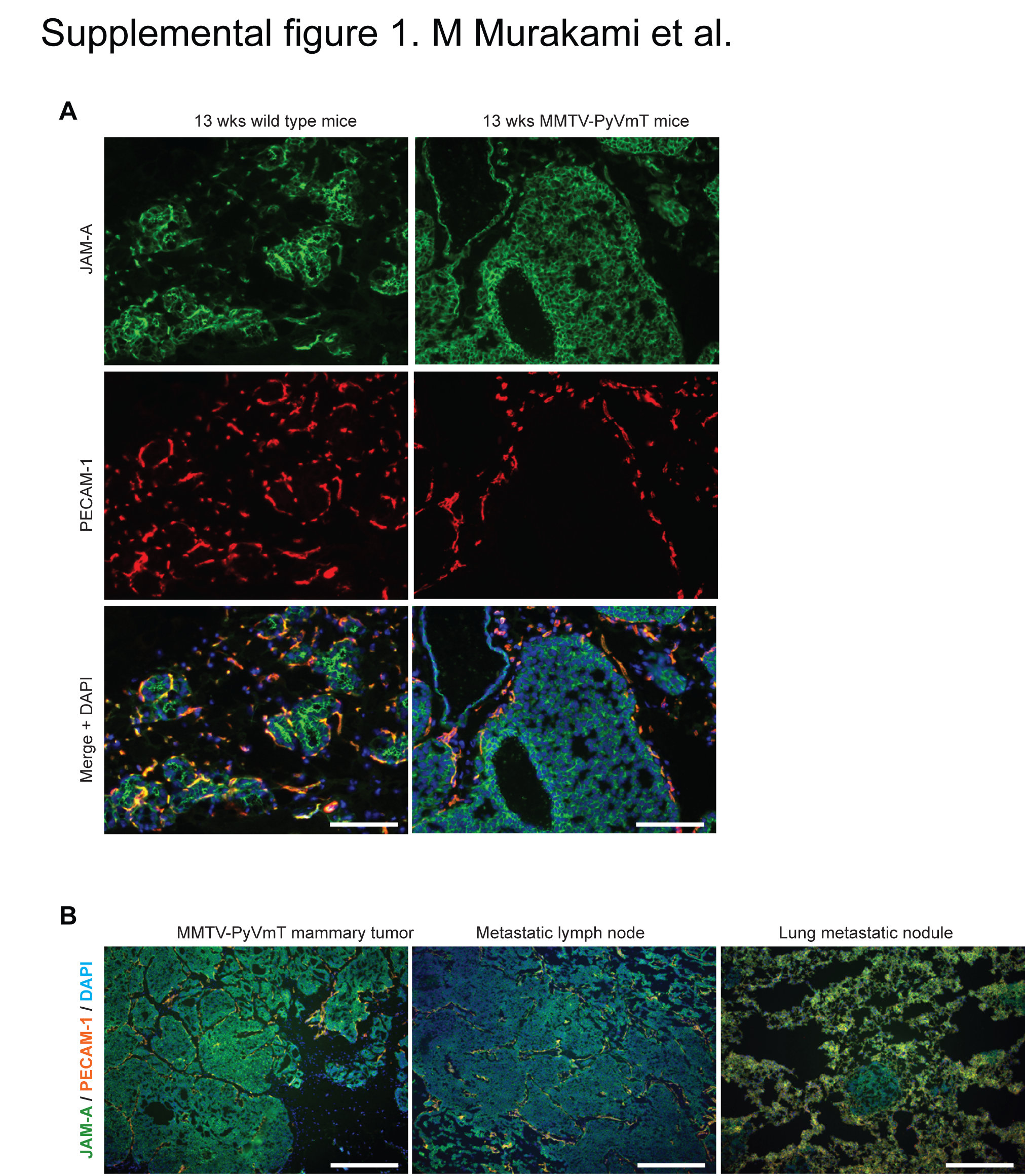

Supplement: Figure S1 — JAM-A is expressed both in wild type and MMTV-PyVmT mice mammary tumors by immunohistological analysis of tissue samples. (A) JAM-A is expressed in epithelial of JAM-A +/+ mammary gland mice (left panel) and not in that of JAM-A −/− mice (right panel). Scale bar 200 µm. (B) JAM-A is expressed in epithelial of normal mammary gland (left panel) and MMTV mammary tumor (right panel). Scale bar 100 µm. (C) JAM-A is expressed in primary mammary tumor, metastatic lymph nodes and metastatic lung tumor. Scale bar 400 µm. (TIF) [file pone.0021242.s001.tif]

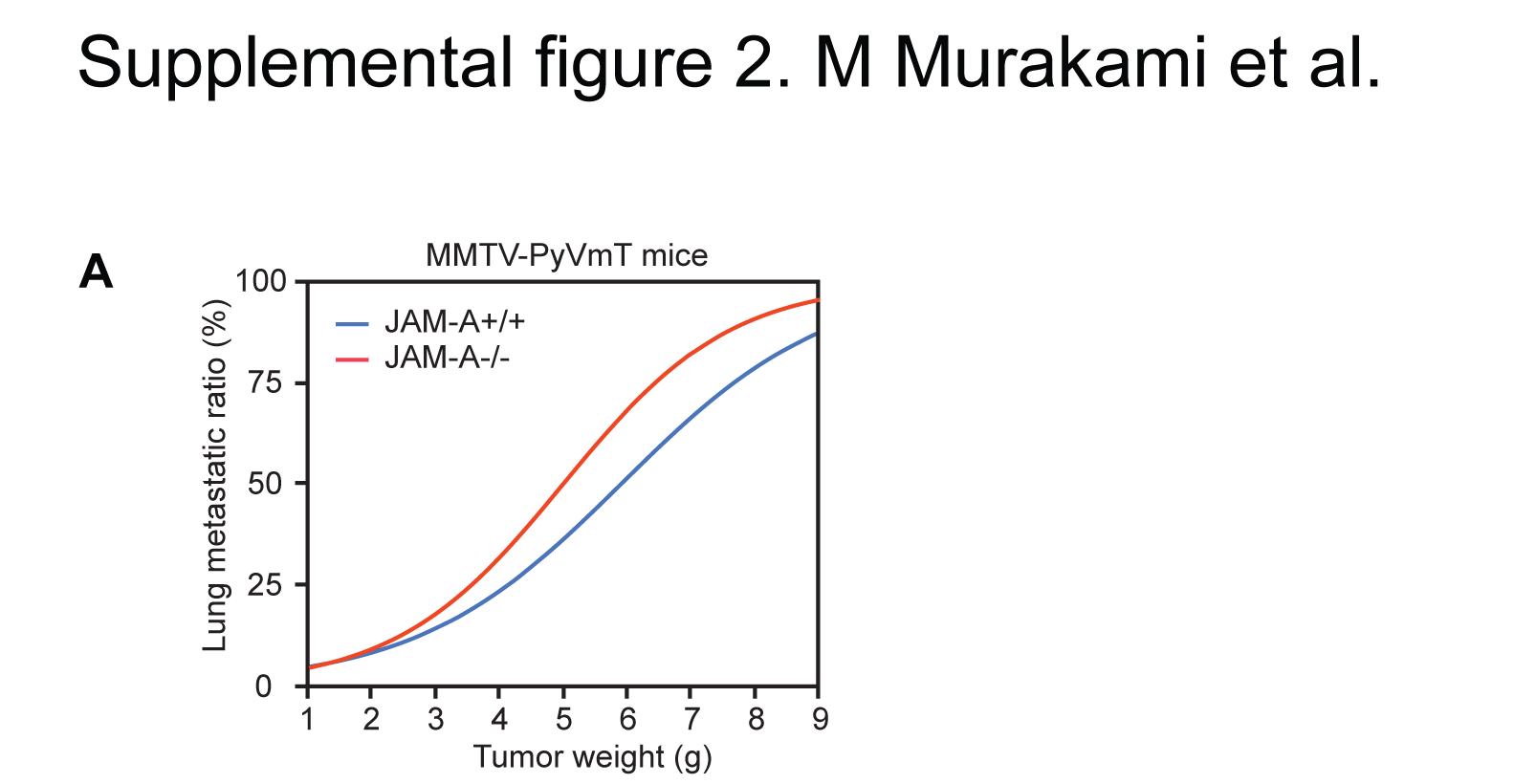

Supplement: Figure S2 — Mammary tumor weight positively correlates with lung metastasis in MMTV-PyVmT mice, but JAM-A expression does not affect this parameter. Relationship between primary mammary tumor size and lung metastases of MMTV mice with or without JAM-A. Primary mammary tumor weight and lung metastases are positively correlated both in MMTV-PyVmT/JAM-A+/+ and in MMTV-PyVmT/JAM-A−/− mice but the lung metastatic ratio show no significant difference between these groups. (TIF) [file pone.0021242.s002.tif]

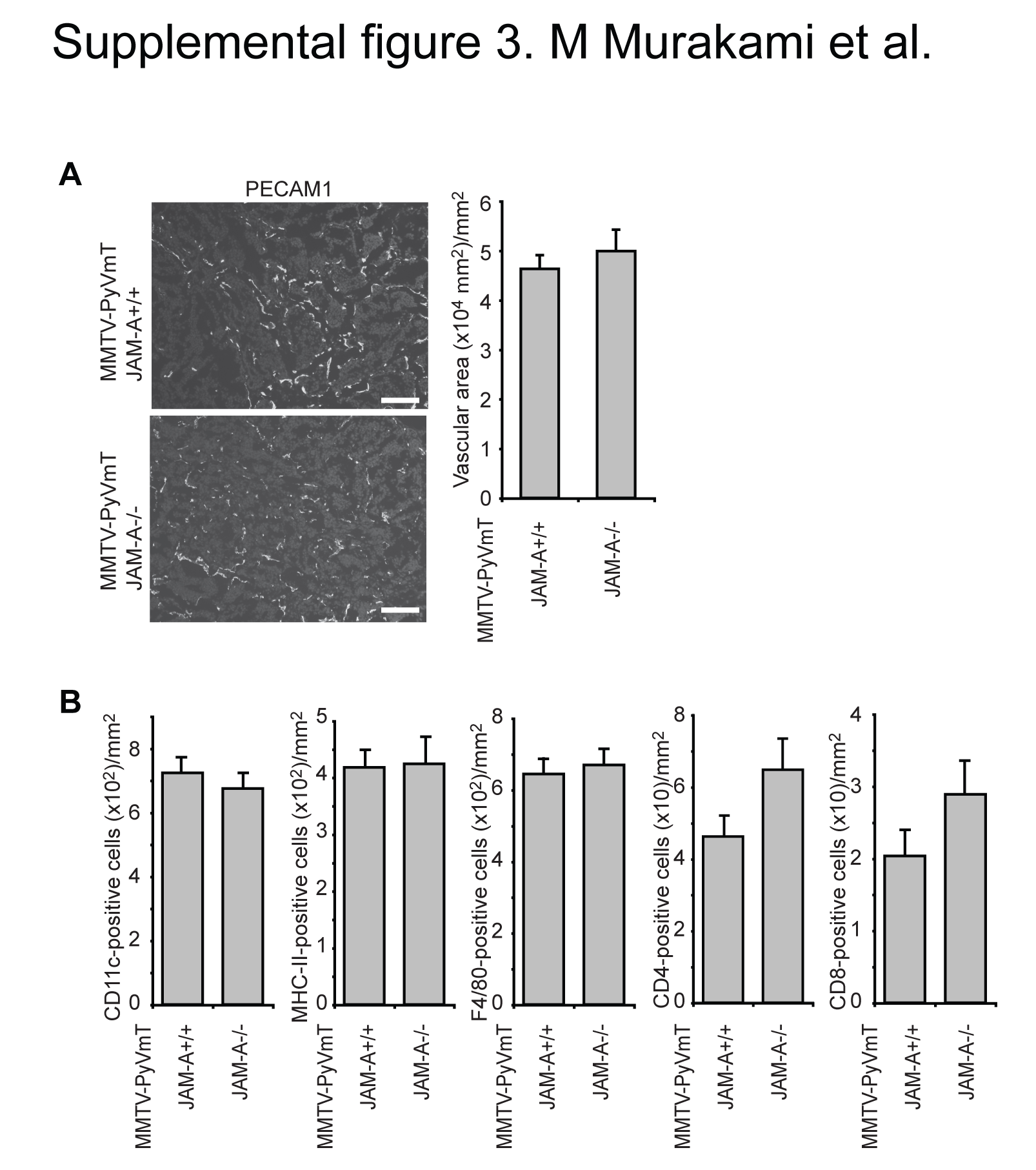

Supplement: Figure S3 — Immunohistological analysis of MMTV-PyVmT/JAM-A−/− or JAM-A+/+ tumors. A) Vascular density evaluated by PECAM-1 staining of vessels was not significantly different in JAM-A +/+ and −/− tumors (46252±2776 versus 49838±4311 µm2/mm2 p = 0.488; means ± SEM) Scale bar; 100 µm. B) No significant difference were observed in infiltration of CD11c- and MHC-II-positive dendritic cells, F4/80-positive macrophages and CD4- and CD8-positive leukocytes in MMTV-PyVmT/JAM+/+ or −/− tumors. (CD11c-positive cells, 723±49.33 vs 674.47±49.08 cells per mm2 p = 0.489; MHC-II-positive cells 417.8±31.12 vs 423.73±48.03 cells per mm2 p = 0.918; F4/80-positive cells 643.73±43.61 vs 669.27±42.40 cells per mm2 p = 0.677; CD4-positive cells 46.2±5.84 vs 64.8±8.58 cells per mm2 p = 0.080; CD8-positive cells 20.33±3.60 vs 28.87±4.69 cells per mm2 p = 0.156. Data represent means ± SEM). (TIF) [file pone.0021242.s003.tif]

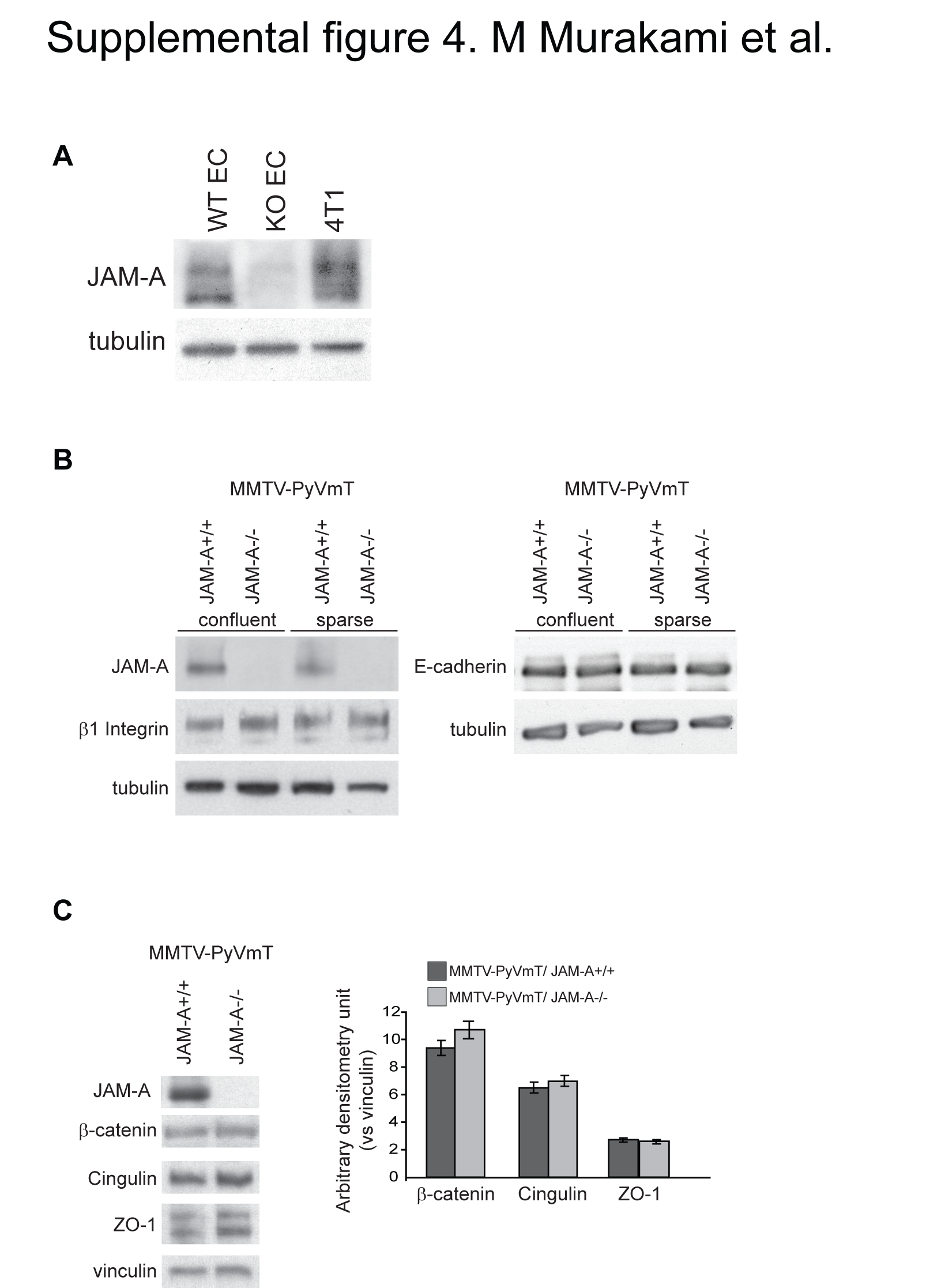

Supplement: Figure S4 — Characterization of mammary cells. A) JAM-A expression in 4T1 cells by Western blot analysis. Data show that 4T1 cells express a comparable level of JAM-A as compared to wild type endothelial cells (EC). The specificity of the Ab (BV11) was indicated by lack of staining of EC from JAM-A−/− mice [18]. B) Western blot analysis shows that cultured MMTV-PyVmT/JAM-A+/+ mammary tumor cells express JAM-A in sparse and confluent conditions. As expected, JAM-A staining was absent in cells derived from tumors of JAM-A null mice. Integrin β1 chain or E-cadherin were not significantly changed in the presence or absence of JAM-A. (TIF) [file pone.0021242.s004.tif]

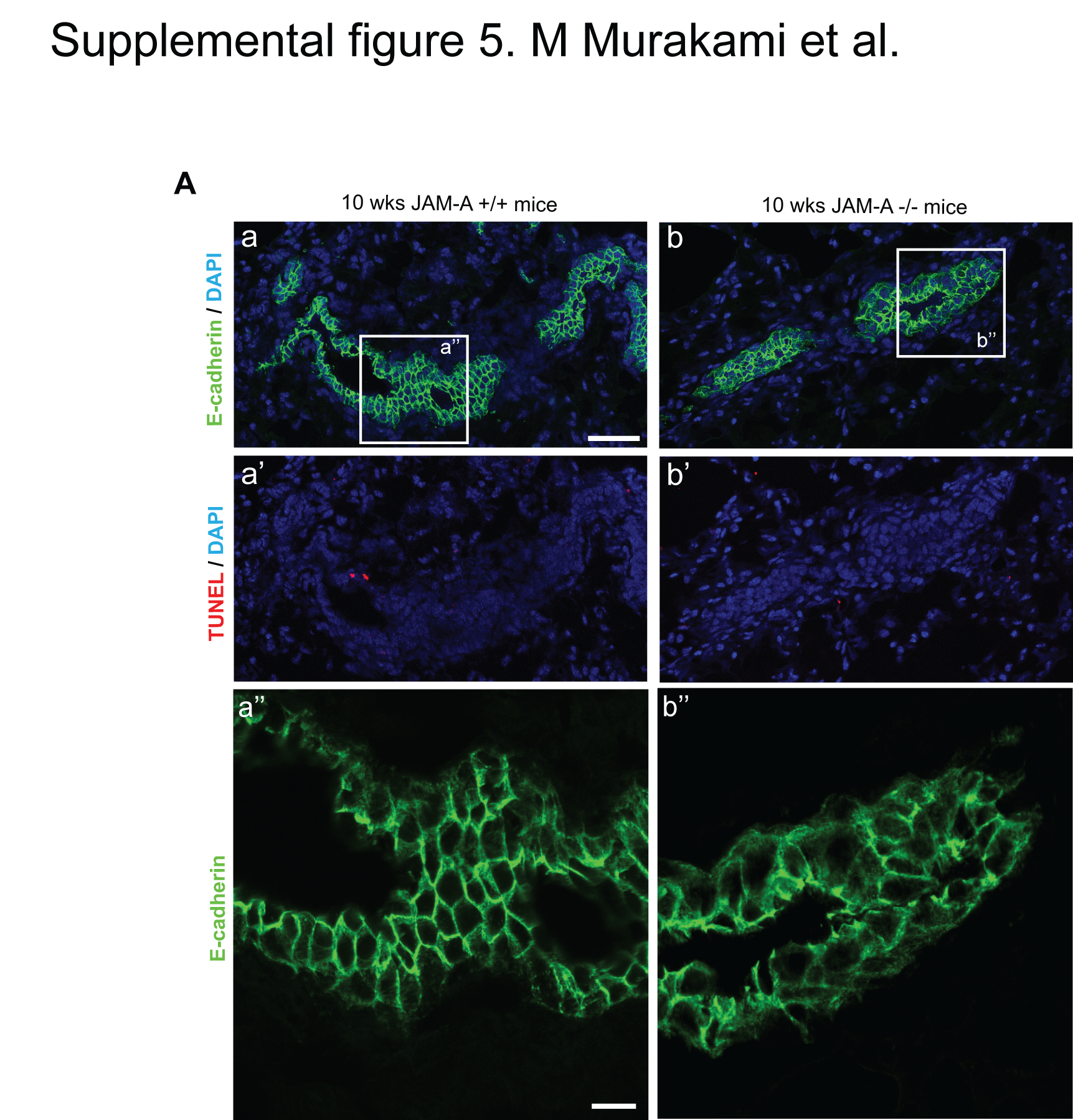

Supplement: Figure S5 — Inhibition of JAM-A expression affects the organization of cell-cell junctions of mammary gland, but does not affect apoptosis. A) Immunofluorescence staining of E-cadherin and TUNEL. While in JAM-A+/+ mammary glands epithelial cells present a continuous junctional staining of E-cadherin at intercellular contacts (a, a″), in JAM-A−/− mammary glands the distribution of this marker was more discontinuous (b, b″). No differences of the TUNEL staining have been observed (a′, b′). Scale bars: 100 µm (a, a′,b, b′) and 10 µm (a″, b″). (TIF) [file pone.0021242.s005.tif]
