# Supplementary material for: Abrogation of Junctional Adhesion Molecule-A Expression Induces Cell Apoptosis and Reduces Breast Cancer Progression
Source: PLoS One. 2011 Jun 17;6(6):e21242. doi: 10.1371/journal.pone.0021242 (PMC3117883; doi:10.1371/journal.pone.0021242)
Supplement: Table S3 — Clinical and pathological information of the case-control dataset of breast cancer patients. The clinical and pathological information of the patients of the case-control dataset is reported. Disease recurrence (all events and distant events) was within 7 years. For some patients not all information was available. Nottingham Prognostic Index (NPI) combines nodal status, tumor size and histological grade. According to NPI's score patients can be divided into 3 classes: Good Prognosis Group (GPG), Moderate Prognosis Group (MPG) and Poor Prognosis Group (PPG). Primary tumor size of WHO classification, pT; Estrogen receptor, ER; Progesterone receptor, PGR. (DOC) [file pone.0021242.s008.doc]

**Supplemental table 3.** *Clinical and pathological information of the case-control dataset of breast cancer patients.*

| **PARAMETER** | **GROUP** | **CASE-CONTROL COHORT (N = 444)** | |
| --- | --- | --- | --- |
|  |  | **N** | **%** |
| **Age** | *< 50* | 207 | 46.7 |
|  | *≥ 50* | 236 | 53.3 |
| **Histotype** | *Ductal* | 363 | 81.9 |
|  | *Lobular* | 70 | 15.8 |
|  | *Other* | 10 | 2.3 |
| **pT** | *1* | 250 | 58.7 |
|  | *2* | 148 | 34.7 |
|  | *3* | 20 | 4.7 |
|  | *4* | 8 | 1.9 |
| **Nodal Status** | *Neg* | 246 | 55.5 |
|  | *Pos* | 197 | 44.5 |
| **GRADE** | *1* | 80 | 21.1 |
|  | *2* | 167 | 44.1 |
|  | *3* | 132 | 34.8 |
| **ER** | *< 10%* | 130 | 30.6 |
|  | *≥ 10%* | 295 | 69.4 |
| **PgR** | *< 10%* | 173 | 42.0 |
|  | *≥ 10%* | 239 | 58.0 |
| **Ki-67** | *< 16%* | 196 | 47.6 |
|  | *≥ 16%* | 216 | 52.4 |
| **ErbB2** | *Low* | 345 | 85.6 |
|  | *High* | 58 | 14.4 |
| **NPI** | *GPG* | 125 | 36.0 |
|  | *MPG* | 139 | 40.1 |
|  | *PPG* | 83 | 23.9 |
| **ALL EVENT (ANY)** | *event* | 211 | 47.5 |
|  | *no event* | 233 | 52.5 |
| **DISTANT EVENT** | *event* | 121 | 27.3 |
|  | *no event* | 323 | 72.7 |
